# Flexible, highly efficient all-polymer solar cells

**DOI:** 10.1038/ncomms9547

**Published:** 2015-10-09

**Authors:** Taesu Kim, Jae-Han Kim, Tae Eui Kang, Changyeon Lee, Hyunbum Kang, Minkwan Shin, Cheng Wang, Biwu Ma, Unyong Jeong, Taek-Soo Kim, Bumjoon J. Kim

**Affiliations:** 1 Department of Chemical and Biomolecular Engineering, Korea Advanced Institute of Science and Technology (KAIST), Daejeon 305-701, Republic of Korea; 2 KI for the NanoCentury, KAIST, Daejeon 305-701, Republic of Korea; 3 Department of Mechanical Engineering, KAIST, Daejeon 305-701, Republic of Korea; 4 Department of Materials Science and Engineering, POSTECH, Pohang 790-784, Republic of Korea; 5 Advanced Light Source, Lawrence Berkeley National Laboratory, Berkeley, California 94720, USA; 6 Department of Chemical and Biomedical Engineering, Florida State University, Tallahassee, Florida 32310, USA

## Abstract

All-polymer solar cells have shown great potential as flexible and portable power generators. These devices should offer good mechanical endurance with high power-conversion efficiency for viability in commercial applications. In this work, we develop highly efficient and mechanically robust all-polymer solar cells that are based on the PBDTTTPD polymer donor and the P(NDI2HD-T) polymer acceptor. These systems exhibit high power-conversion efficiency of 6.64%. Also, the proposed all-polymer solar cells have even better performance than the control polymer-fullerene devices with phenyl-C_61_-butyric acid methyl ester (PCBM) as the electron acceptor (6.12%). More importantly, our all-polymer solar cells exhibit dramatically enhanced strength and flexibility compared with polymer/PCBM devices, with 60- and 470-fold improvements in elongation at break and toughness, respectively. The superior mechanical properties of all-polymer solar cells afford greater tolerance to severe deformations than conventional polymer-fullerene solar cells, making them much better candidates for applications in flexible and portable devices.

## Abstract (web summary)

All-polymer solar cells have advantages over fullerene-based solar cells due to improved stability and tunable chemical and electronic properties. Here, Kim *et al.* develop highly efficient and robust solar cells based on PBDTTTPD and P(NDI2HD-T), highlighting their potential in flexible and portable electronics.

## Graphical abstract

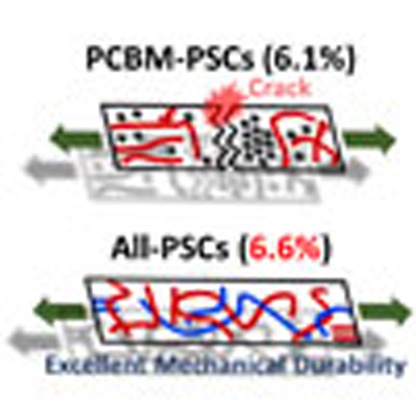

The emergence of flexible and wearable devices, such as electronic textiles, smart watches and patchable sensors, has stimulated research on alternative power generators for operating such devices. One of the basic requirements for these devices is that the integration of individual electronic components must retain flexibility and portability to function in new operational environments^1^,^2^,^3^,^4^. For this consideration, organic solar cells (OSCs) are currently viewed as promising power generation technologies that can be integrated into these devices because they are lightweight, semitransparent and flexible^5^,^6^. To date, most highly efficient OSCs have been based on polymer-fullerene blends, in which fullerenes such as phenyl-C_61_-butyric acid methyl ester (PCBM) act as the electron acceptor. However, fullerenes are not ideal acceptor materials due to many intrinsic issues, such as weak light absorption and un-optimized energy levels, limiting the design adaptability of the electron donor pair. Moreover, fullerene-based OSCs have low flexibility and stretchability due to the brittle crystalline features of the fullerenes^7^,^8^. To resolve these drawbacks, a number of non-fullerene acceptors, including small molecules, nanoparticles and polymers, have been developed to replace fullerenes^9^,^10^,^11^,^12^.

All-polymer solar cells (all-PSCs), consisting of polymer-donor and polymer-acceptor materials, possess many advantages over polymer-fullerene solar cells, including tunable chemical and electronic properties as well as enhanced stabilities^13^,^14^,^15^,^16^,^17^,^18^,^19^,^20^,^21^. In recent years, many efforts have focused on developing optimal polymer-donor and polymer-acceptor combinations with well-controlled bulk-heterojunction (BHJ) morphologies^21^,^22^,^23^,^24^,^25^,^26^,^27^,^28^,^29^. Among the non-fullerene acceptors, naphthalene diimide (NDI)-based copolymers have been the most successful polymer acceptors with high electron affinities and high electron mobilities, which are a result of their highly extended *π*-conjugated structure and strong *π*–*π* intermolecular interaction^30^,^31^,^32^,^33^,^34^,^35^. The power-conversion efficiencies (PCEs) of all-PSCs have improved greatly to 5–6%, and there is still great potential for further enhancement^26^,^27^,^37^,^38^, provided that simultaneous adjustment of polymer-donor and polymer-acceptor energy levels can improve their light harvesting and increase the open-circuit voltage (*V*_OC_). Compared with conventional polymer-fullerene solar cells, all-PSCs can potentially exhibit much better mechanical strength and stability, because polymer acceptors are not only intrinsically more ductile than fullerenes but also are entangled with other polymers within the acceptor domain and at the interface^39^,^40^,^41^. In consideration of the application of PSCs in flexible devices, the mechanical properties of the all-PSCs should be investigated. However, to our knowledge, there is no report regarding this important topic.

Herein, we demonstrate that all-PSCs are better candidates than fullerene-based solar cells for applications in flexible and portable electronics. Highly efficient and mechanically robust all-PSCs have been fabricated by using poly[4,8-bis(5-(2-ethylhexyl)thiophen-2-yl)benzo[1,2-b:4,5-b′]dithiophene-*alt*-1,3-bis(thiophen-2-yl)-5-(2-hexyldecyl)-4*H*-thieno[3,4-c]pyrrole-4,6(5*H*)-dione](PBDTTTPD) as the electron donor^42^,^43^,^44^ and poly[[*N*,*N*′-bis(2-hexyldecyl)-naphthalene-1,4,5,8-bis(dicarboximide)-2,6-diyl]-*alt*-5,5′-thiophene] (P(NDI2HD-T)) as the electron acceptor. The all-PSCs based on this donor/acceptor pair of PBDTTTPD and P(NDI2HD-T) exhibited a PCE of 6.64%, which is higher than that of PBDTTTPD:PCBM BHJ PSCs (PCE=6.12%). The better performance of the all-PSCs was attributed to the high *V*_OC_ of 1.06 V and the optimized BHJ active layers of polymer donor and acceptor with favourable interfacial interactions. We have also studied the mechanical properties of the all-PSCs and found that, compared with fullerene-based blend films, all-polymer blend films offer superior flexibility, stretching and bending properties.

## Results

### Photovoltaic performances

**Figure 1:**
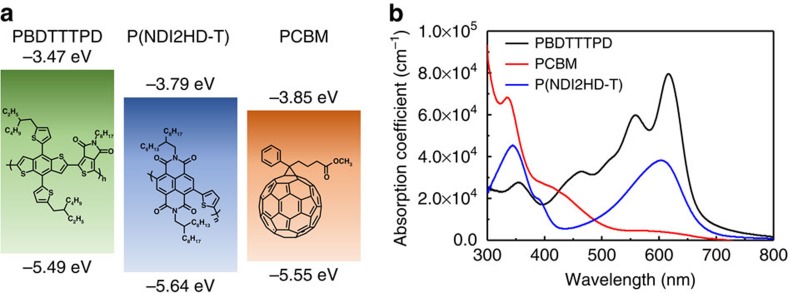
Polymer information. (**a**) Chemical structures, energy levels, and (**b**) ultraviolet-visible absorption spectra of PBDTTTPD (black line), PCBM (red line) and P(NDI2HD-T) (blue line).

**Figure 2:**
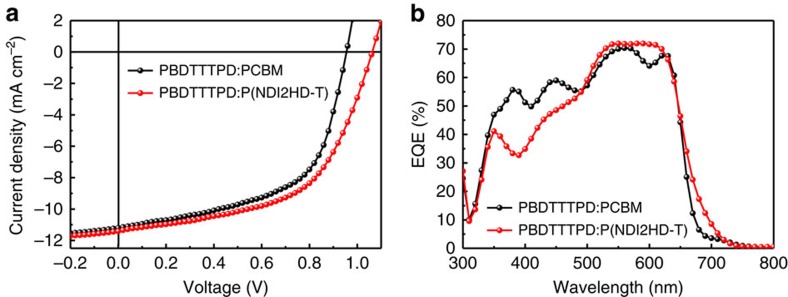
*J–V* and EQE characteristics for the PCBM-PSC and the all-PSC. (**a**) *J*–*V* curves of normal-type devices, PBDTTTPD:PCBM (black line), PBDTTTPD:P(NDI2HD-T) (red line) under AM 1.5 G-simulated solar illumination (100 mW cm^−2^); (**b**) EQE characteristics of the PBDTTTPD:PCBM (black line) and PBDTTTPD:P(NDI2HD-T) (red line).

Figure 1a shows the molecular structures and energy levels of PBDTTTPD, P(NDI2HD-T) and PCBM. The PBDTTTPD had number-average molecular weight (*M*_n_) value of 22 kg mol^−1^ and optical bandgap of 2.02 eV (see Supplementary Table 1), absorbing light in the wavelength of 400–650 nm (Fig. 1b). The P(NDI2HD-T) was also synthesized with *M*_n_ value of 48 kg mol^−1^, after considering that (1) the P(NDI2HD-T) had a higher lowest unoccupied molecular orbital energy level than that of PCBM, and (2) it had high electron mobility^26^. To explore the potential of these polymers in a photovoltaic system, we fabricated all-PSCs using a blend of PBDTTTPD and P(NDI2HD-T), and compared their performance with conventional polymer-fullerene solar cells (PCBM-PSCs) based on a blend of PBDTTTPD and PCBM. Both PSCs were fabricated with the same device structure of ITO/poly-(3,4-ethylenedioxythiophene):poly(styrenesulfonate) (PEDOT:PSS)/blend layer/LiF/Al, although the processing conditions for the optimized PBDTTTPD:PCBM and PBDTTTPD:P(NDI2HD-T) blend layers were slightly different. For the PBDTTTPD:PCBM system, we used chloroform and 1,8-diiodooctane (DIO, 3 vol%) as the processing solvents, and a polymer-donor concentration of 10 mg ml^−1^ with the donor:acceptor ratio of 1:1.5 (w/w). For the PBDTTTPD:P(NDI2HD-T) system, chloroform with 1 vol% of DIO was used to process a 12.5 mg ml^−1^ blend solution (1.3:1, w/w). The optimized film thicknesses of the PBDTTTPD:PCBM and PBDTTTPD:P(NDI2HD-T) blend layers were ∼200 and 130 nm, respectively. Details about the device optimization can be found in Supplementary Tables 2, 3 and 4. Figure 2 shows the current density versus voltage (*J*–*V*) curves and external quantum efficiency (EQE) spectra of the optimized PSCs, and Table 1 summarizes the corresponding photovoltaic characteristics. The best PCE of the PCBM-PSCs was 6.12% (*V*_OC_=0.96 V; *J*_SC_=11.17 mA cm^−2^; fill factor (FF)=0.57). This PCE value was consistent with or even higher than the PCE values of 4–6% reported in the literature for the PBDTTTPD:PCBM systems^42^,^43^,^44^. In contrast, when the P(NDI2HD-T) polymer was used as the electron acceptor, the best PCE increased significantly to 6.64% with *V*_OC_ of 1.06 V, which represents one of the highest PCE and *V*_OC_ values reported to date for all-PSCs. The higher device performance of all-PSCs was mainly attributed to the enhanced *V*_OC_ value because of the higher-lying lowest unoccupied molecular orbital energy level of P(NDI2HD-T) than PCBM (Fig. 1a). We also measured the EQE spectra for the optimized PCBM-PSCs and all-PSCs. The *J*_SC_ values were well matched (within 3% error) with the integrated *J*_SC_ values obtained from the EQE spectra (Fig. 2b and Table 1). The EQE values of all-PSCs were higher than those of PCBM-PSC in the low-energy region of 500–700 nm, but lower in the high-energy region of 350–500 nm. This was not surprising if we considered the difference of the absorption for P(NDI2HD-T) and PCBM, that is, P(NDI2HD-T) having higher absorbance in the low-energy region but lower absorbance in the high-energy region compared with PCBM (Supplementary Fig. 1). Overall, comparable values of *J*_SC_ were obtained for both PSCs.

### Polymer packing structure and blend morphology

To gain a deeper insight into the photovoltaic operation of the devices, we investigated the morphological characteristics of PBDTTTPD:PCBM and PBDTTTPD:P(NDI2HD-T) blends. First, we examined the polymer packing structures of PBDTTTPD:PCBM and PBDTTTPD:P(NDI2HD-T) blends via grazing incidence X-ray scattering (GIXS) measurements (Supplementary Fig. 2). Supplementary Fig. 2a shows that the PBDTTTPD and P(NDI2HD-T) neat films had (100) scattering peaks in the in-plane direction (*q*_xy_) with lamellar domain spacings of 24.1 Å (*q*_in_=0.26 Å^−1^) and 22.5 Å (*q*_in_=0.28 Å^−1^) and these scattering features were well preserved in the blend films (see Supplementary Fig. 2b). Prominent (010) peaks of PBDTTTPD:PCBM and PBDTTTPD:P(NDI2HD-T) blends, corresponding to *π*–*π* stacking, were shown in the out-of-plane direction, indicating that both blends strongly preferred a face-on orientation relative to the substrate. The face-on stacked polymer blends should be beneficial for charge transport through the active layer to the electrodes^18^,^45^,^46^. Second, the blend morphologies of the PBDTTTPD:PCBM and PBDTTTPD:P(NDI2HD-T) films were characterized by atomic force microscopy (AFM) and resonant soft X-ray scattering (RSoXS) measurements (Supplementary Fig. 3). The AFM images showed that the PBDTTTPD:PCBM blend had coarser domains with a much larger surface roughness (root-mean-square value of 6.4 nm) than that of the PBDTTTPD:P(NDI2HD-T) blend (3.1 nm). In addition, the RSoXS measurements showed that the scattering peak of the PBDTTTPD:P(NDI2HD-T) film had larger *q* value (0.0097 Å^−1^) with much weaker intensity compared with that of PBDTTTPD:PCBM blend film (*q*=0.0036 and 0.0083 Å^−1^), suggesting that PBDTTTPD:P(NDI2HD-T) blends possessed smaller domain size and much better intermixing^26^,^47^,^48^,^49^. The trend of RSoXS measurements was consistent with the results of the AFM measurements. The well-intermixed BHJ morphologies with smaller phase-separated domains for the PBDTTTPD:P(NDI2HD-T) blend could be partly attributed to the relatively low value of the interfacial tension (*γ*) between the polymer donor and the polymer acceptor, which was estimated by the contact angle measurements (Supplementary Fig. 4 and Supplementary Table 5)^50^,^51^.

### Space charge limited current charge mobility

We have also evaluated the hole mobility (*μ*_h_) and electron mobility (*μ*_e_) of the PBDTTTPD:PCBM and PBDTTTPD:P(NDI2HD-T) blends by using the space charge limited current measurements (Table 1 and Supplementary Fig. 5). The *μ*_h_ values of the PCBM-based and the all-polymer-based blends were almost same, that is, 2.5 × 10^–5^ and 2.8 × 10^–5^ cm^2^ V^−1^ s^−1^, respectively. The *μ*_h_ value of the PBDTTTPD:PCBM film was consistent with that reported previously^42^. Both of the blends had *μ*_e_ values with the same order of magnitude, that is, 10^–5^ cm^2^ V^−1^ s^−1^, with the *μ*_e_ value of the PBDTTTPD:PCBM being higher. A *μ*_h_/*μ*_e_ value of 1.8 was obtained for the PBDTTTPD:P(NDI2HD-T) film, suggesting greatly more balanced hole and electron transports than those of other high-performance all-PSC systems^17^,^21^. Overall, the all-PSC system possessed desired features of polymer packing structure, blend morphology, and electrical properties, to achieve high performance, which were indeed better than or comparable to those of the fullerene-based PSC system.

### Mechanical properties

The excellent mechanical stability of all-PSCs represents another major merit for their potential applications in the portable and outdoor devices^52^,^53^,^54^. The sharp and weak interfaces between the polymer/fullerene junctions of the fullerene PSCs result in low cohesion and poor ductility in the BHJ active layer, and consequently mechanical fragility of the entire device^7^,^54^,^55^,^56^,^57^. In addition, the fullerenes in the blend film have strong tendency to crystallize, making the film even stiffer and more brittle with higher tensile moduli and lower cohesive energy with polymer donors^8^. Compared with PCBM-PSCs, all-PSCs are expected to have much better mechanical properties owing to (i) the polymer acceptor's greater intrinsic flexibility than fullerenes, and (ii) the strengthened donor/acceptor interfaces by the entanglements between the polymer chains^39^,^58^.

**Figure 3:**
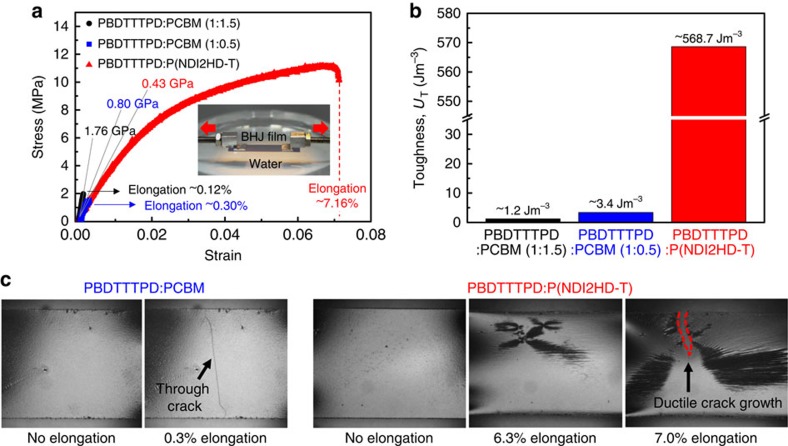
Tensile test of PBDTTTPD:PCBM and PBDTTTPD:P(NDI2HD-T) blend films. (**a**) Strain–stress curves and (**b**) toughness of PBDTTTPD:PCBM and PBDTTTPD:P(NDI2HD-T) blend films. (The inset in **a** shows photographs of the BHJ blend film floating on water. The specimens were gripped by the PDMS-coated Al grips and the films were prepared under the optimized device condition). (**c**) Optical microscopy images of PBDTTTPD:PCBM (1:0.5 w/w) and PBDTTTPD:P(NDI2HD-T) (1.3:1 w/w) blend films when the films were under different strains.

To illustrate the difference of mechanical properties between PCBM-PSCs and all-PSCs, we first measured the tensile characteristics of PBDTTTPD:PCBM and PBDTTTPD:P(NDI2HD-T) blend films to obtain a quantitative comparison of the mechanical resilience of the two films (Fig. 3). We conducted a ‘pseudo free-standing tensile test' (Supplementary Fig. 6), in which the PBDTTTPD:PCBM and PBDTTTPD:P(NDI2HD-T) thin films without any substrate were floated on the water surface without any significant damage to or wrinkling of the specimens^56^,^59^. Therefore, the intrinsic mechanical properties of the films, including tensile modulus and elongation at break, were directly measured without any substrate effects, complex calculations or assumptions. Figure 3a shows the stress–strain curves of the PBDTTTPD:PCBM and PBDTTTPD:P(NDI2HD-T) blend films. The elastic modulus and the elongation at break of the PBDTTTPD:PCBM (1:1.5 w/w) blend film were measured to be 1.76 GPa and 0.12%, respectively. In addition to the optimized device condition of PBDTTTPD:PCBM (1:1.5 w/w), we also performed a tensile test for the PBDTTTPD:PCBM blend with different blend ratio (1:0.5 w/w) and obtained a similar tensile modulus of 0.80 GPa and an elongation at break of 0.30%. The observed brittleness decreased as the PCBM content was lowered, which is consistent with what was previously reported and suggested that PCBM is the performance limiting component^7^,^60^. In contrast, and surprisingly, the tensile modulus of the PBDTTTPD:P(NDI2HD-T) blend film was only 0.43 GPa, and its elongation at break of PBDTTTPD:P(NDI2HD-T) blend was 7.16%, which was a 60-fold enhancement over that of PBDTTTPD:PCBM. Note that considering the applications of all-PSCs in the wearable devices, this high value of elongation at break of our all-PSC satisfies the requirements^61^,^62^. These superior mechanical characteristics of all-PSC film than that of the PCBM-based BHJ film can provide a clear advantage for flexible and even stretchable electronic applications that require high tolerance against severe mechanical deformations. It should be noted that this is the first demonstration of all-PSC films with excellent mechanical resilience.

The excellent mechanical stability of the all-polymer films was also clearly confirmed by the calculation of toughness (Fig. 3b). The toughness of the BHJ films was obtained from the integration of the stress–strain curves in Fig. 3a. We found a remarkable contrast in the toughness values of the all-PSC films (568.7 J m^−3^) and the PCBM-PSC films (1.2 J m^−3^); the toughness of the all-PSC film was 470 times greater than that of the PCBM-PSC film. The tensile behaviours of the all-PSC film and PCBM-based film (1:0.5 w/w) were compared using an optical microscopy during the tensile tests (Fig. 3c). The results indicated that there was a dramatic difference in their fracture responses under tensile strain. The PCBM-based film had a sharp and through crack at very low value of elongation (0.3%), representing its brittle nature. In contrast, the all-polymer film had a ductile nature, exhibiting no crack until high elongation of 7%. This significantly improved mechanical property of the PBDTTTPD:P(NDI2HD-T) blend film can be attributed primarily to the ductility of the polymer films imposed by the entangled polymer chains^39^,^40^. In addition, the optimized BHJ morphologies of the PBDTTTPD:P(NDI2HD-T) blend with large interfacial area and well-intermixed polymer domains may contribute to the enhancement of their mechanical resilience^63^. The outstanding mechanical characteristics of the all-PSC films can enhance the ductility and the endurance against mechanical deformations by effectively relieving stress without mechanical failure, which is a critical requirement for flexible PSCs^52^,^53^.

**Figure 4:**
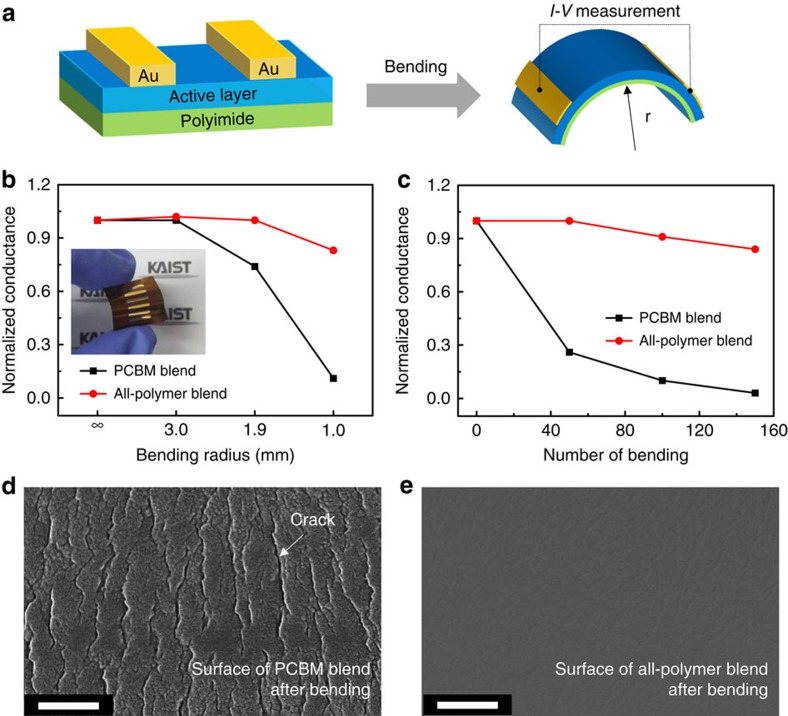
Bending test of PBDTTTPD:PCBM and PBDTTTPD:P(NDI2HD-T) blend films. (**a**) Scheme of the BHJ blend film deposited on the flexible plastic substrate under mechanical bending. Normalized conductance of PBDTTTPD:PCBM and PBDTTTPD:P(NDI2HD-T) blend films (**b**) after bending at various *r* values and (**c**) after multiple cycles of bending at *r*=1.5 mm. SEM images of surface morphologies of (**d**) PBDTTTPD:PCBM and (**e**) PBDTTTPD:P(NDI2HD-T) blend films after bending at *r*=1.0 mm. The scale bars are 500 nm.

To further explore the potential of using all-PSCs in flexible devices, we measured the bending characteristics^3^,^64^,^65^ of the PBDTTTPD:PCBM and PBDTTTPD:P(NDI2HD-T) blend films. Their current–voltage (*I–V*) characteristics were measured (Supplementary Fig. 7), and we obtained normalized conductance [(Δ*I/*Δ*V)/*(Δ*I/*Δ*V*)_0_] by monitoring the change in the Δ*I/*Δ*V* values after bending to examine the stability of their electrical performances against external mechanical deformation (Fig. 4a). The (Δ*I/*Δ*V*)_0_ is conductance value of the film before any bending. The blend films were prepared on a flexible polyimide substrate with a thickness of 80 μm. A 70-nm-thick Au electrode was thermally evaporated onto the films and the distance between the electrodes was 1 mm. Figure 4b compares the normalized conductance of the PBDTTTPD:PCBM and PBDTTTPD:P(NDI2HD-T) blend films after the bending test at different bending radii (*r*=∞, 3.0, 1.9 and 1.0 mm). The electrical property of the fullerene-PSC film was reduced considerably at *r*=1.0 mm, compared with the reference sample at *r*=∞. The apparent degradation was attributed to the crack propagation in the fullerene-PSC film by the mechanical deformation (Fig. 4d). In stark contrast, there was no change in the electrical and morphological properties of the all-PSC film, even at very small *r* value of 1.0 mm (Fig. 4e). In addition, as shown in Fig. 4c, we compared the normalized conductance of the PBDTTTPD:PCBM and PBDTTTPD:P(NDI2HD-T) films after multiple cycles (N=0, 50, 100 and 150) of bending at fixed *r*=1.5 mm. As N increased, the PBDTTTPD:PCBM blend film underwent a massive decrease of the current. However, the conductance of the all-PSC film was very stable, with only negligible changes at the same measurement conditions. The trend of the bending test fully corresponded to the results of the tensile modulus test, and the mechanical stability experiments consistently led to the same conclusion: the mechanical durability of all-PSCs is far superior to that of fullerene PSCs.

In summary, we have demonstrated highly efficient and mechanically robust all-PSCs. By using PBDTTTPD as the electron donor and P(NDI2HD-T) as the electron acceptor, all-PSCs with high PCE of 6.64% have been achieved, which is even higher than that of control fullerene PSCs (PCE=6.12%). The enhanced performance of all-PSCs is mainly attributed to the high *V*_OC_ (1.06 V) due to the better alignment of energy levels. Also playing significant roles are the enhanced absorption of P(NDI2HD-T) in the region of 500–700 nm, as well as the desired BHJ morphology of polymer blends with effective exciton dissociation and charge transport. More importantly, our study has shown that the all-PSCs have far superior mechanical durability compared with that of fullerene PSCs: the elongation at break and the toughness for all-PSCs are over 60 times and 470 times higher than those of the fullerene PSCs, respectively. This is due to the fact that the polymer acceptor is intrinsically more ductile than PCBM and can better entangle with other polymer chains with strengthened interfaces. And the superior electronic and mechanical performances of all-PSCs would make them more suitable for applications in flexible and portable devices than conventional polymer-fullerene PSCs. Our results provide guidelines for the design of new material systems for high-performance all-PSCs and demonstrate their potential for future applications in portable and wearable devices that require both high performances and mechanical stability.

## Methods

### Characterizations

Ultraviolet-visible absorption spectra were obtained using a UV-1800 spectrophotometer (Shimadzu Scientific Instruments) at room temperature. Static contact angles for water and glycerol were measured using contact angle analyzer (Pheonix 150, SEO, Inc.) equipped with a microsyringe that can dispense liquid droplets. AFM measurements were performed using a Veeco Dimension 3,100 instrument in tapping mode. The samples were prepared by spin coating onto PEDOT:PSS/ITO glasses. RSoXS measurements were performed at BL 11.0.1.2 in the Advanced Light Source (USA) using a series of photon energies to determine the maximum scattering contrast between the donor and the acceptors. RSoXS samples were prepared on a PEDOT:PSS/glass substrate under same optimized active layer condition. Then, the active layers were floated on water and transferred to a 1.0 × 1.0 mm, 100-nm-thick Si_3_N_4_ membrane supported by a 5 × 5 mm, 200-μm-thick Si frame (Norcada Inc.). GIXS measurements were performed at beamline 3C in the Pohang Accelerator Laboratory (South Korea). GIXS samples were prepared by spin coating onto a PEDOT:PSS/Si substrates. X-rays with a wavelength of 1.1179 Å were used. The incidence angle (∼0.12°) was chosen to allow for complete penetration of X-rays into the film.

### Device fabrication and measurement

The PCBM-PSCs and all-PSCs were fabricated with an indium tin oxide (ITO)/PEDOT:PSS/(PBDTTTPD:PCBM (or PBDTTTPD:P(NDI2HD-T))/LiF/Al structure. ITO-coated glass substrates were subjected to ultrasonication in acetone, followed by extensive rinsing with deionized water and they were treated with ultrasonication in isopropyl alcohol. The substrates were then dried for several hours in an oven at 80 °C. The ITO substrates were treated with ultraviolet–ozone before PEDOT:PSS deposition. A filtered dispersion of PEDOT:PSS in water (PH 500) was applied by spin coating at 3,000 r.p.m. for 40 s and baking for 20 min at 150 °C in air. After application of the PEDOT:PSS layer, all subsequent procedures were performed in a glove box under an N_2_ atmosphere. Then, each active blending solution was spin-cast onto an ITO/PEDOT:PSS substrate at 1,000 r.p.m. for 60 s (or at 3,000 r.p.m. for 40 s). (Detailed preparation of the active layer solutions is described in the Supplementary Methods). The substrates were then placed in an evaporation chamber and held under high vacuum (<10^−6^ Torr) for more than 1 h before evaporating ∼0.9 nm of LiF and 100 nm of Al. The configuration of the shadow mask produced four independent devices on each substrate. The active area of the fabricated device was 0.09 cm^2^, which was carefully measured by optical microscope. The *J–V* characteristics of the devices were measured under simulated AM 1.5G solar irradiation (100 mW cm^−2^, Peccell: PEC-L01) at ambient condition. This solar simulator system satisfied the class AAB, ASTM standards. The intensity of the solar simulator was calibrated carefully by using a standard silicon reference cell with a KG-5 visible colour filter. The *J–V* behavior was collected using a Keithley 2400 SMU. The EQE results were obtained using a spectral measurement system (K3100 IQX, McScience Inc.). This system applied monochromatic light from a xenon arc lamp at 300 W filtered by a monochromator (Newport) and an optical chopper (MC 2000 Thorlabs) at ambient conditions. The EQE data were obtained under dark conditions. The theoretical *J*_SC_ values were acquired by integrating the product of the EQE with the AM 1.5 G solar spectrum and they were in good agreement with the measured *J*_SC_ to within 3% error.

### Pseudo free-standing tensile test

For the tensile testing specimen, the active layers were spin-coated onto the PEDOT:PSS/glass substrate. The active layer specimen with a size of 2.54 × 0.5 cm was prepared by using a cutting plotter (GCC Jaguar IV-61, USA). To float the specimen on the water surface, water was allowed to penetrate into the PEDOT:PSS layer. Subsequently, PEDOT:PSS was dissolved, and the active layer was delaminated from the glass substrate. By performing this process at the water surface, the floating active layer specimen could be obtained. Specimen gripping was achieved by attaching PDMS-coated Al grips on the specimen gripping areas using van der Waals adhesion. The tensile test was performed by a linear stage with a strain rate of 0.06 × 10^–3^ s^−1^. During the tensile test, stress and strain data were obtained through a load cell (LTS-10GA, KYOWA, Japan) and a digital image correlation (DIC) device, respectively.

### Bending test

The *I–V* curves were measured by a probe-station system (MST 8000C, HP 4156A).

## Additional information

**How to cite this article:** Kim, T. *et al.* Flexible, highly efficient all-polymer solar cells. *Nat. Commun.* 6:8547 doi: 10.1038/ncomms9547 (2015).

## Supplementary Material

Supplementary Information Supplementary Figures 1-7, Supplementary Tables 1-5, Supplementary Methods and Supplementary References.

## Figures and Tables

**Table 1:** Photovoltaic characteristics and hole and electron mobility values of PBDTTTPD:PCBM and PBDTTTPD:P(NDI2HD-T) systems.

**Device type**	***V***_**OC**_ **(V)**	***J***_**SC**_ **(mA cm**^−**2**^**)**	**FF**	**PCE**_max_* **(PCE**_**ave**_**)**† **(%)**	***J***_**SC**_ **(EQE)**‡ **(mA cm**^−**2**^**)**	***μ***_**h**_ **(cm**^**2**^ **V**^−**1**^ **s**^−**1**^**)**	***μ***_**e**_ **(cm**^**2**^ **V**^−**1**^ **s**^−**1**^**)**	***μ***_**h**_**/*****μ***_**e**_
PCBM-PSCs	0.96 (0.959±0.003)	11.17 (11.208±0.057)	0.57 (0.565±0.007)	6.12 (6.076±0.045)	10.89	2.52 × 10^−5^	6.40 × 10^−5^	0.4
All-PSCs	1.06 (1.062±0.001)	11.22 (11.243±0.028)	0.56 (0.553±0.006)	6.64 (6.601±0.058)	10.96	2.84 × 10^−5^	1.55 × 10^−5^	1.8

^*^Photovoltaic characteristics obtained under AM 1.5 G-simulated solar illumination (100 mW cm^−2^).

^†^The average values were obtained from at least 12 devices.

^‡^Integrated values obtained from the EQE spectra.
