# Supplementary material for: Flexible, highly efficient all-polymer solar cells
Source: Nat Commun. 2015 Oct 9;6:8547. doi: 10.1038/ncomms9547 (PMC4633811; doi:10.1038/ncomms9547)
Supplement: Supplementary Information — Supplementary Figures 1-7, Supplementary Tables 1-5, Supplementary Methods and Supplementary References. [file ncomms9547-s1.pdf]

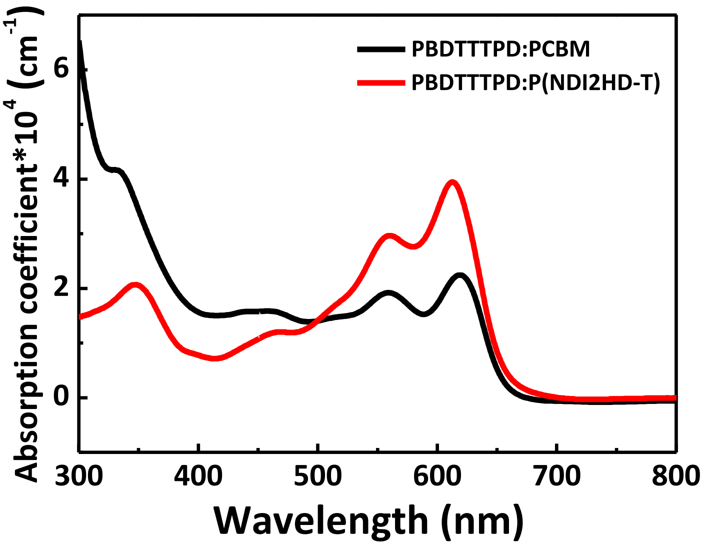

Supplementary Figure 1. UV-vis absorption characteristics of BHJ blend films. UV-vis absorption spectra of PBDTTTPD:PCBM (1:1.5, w/w) and PBDTTTPD:P(NDI2HD-T) (1.3:1, w/w) BHJ blend films

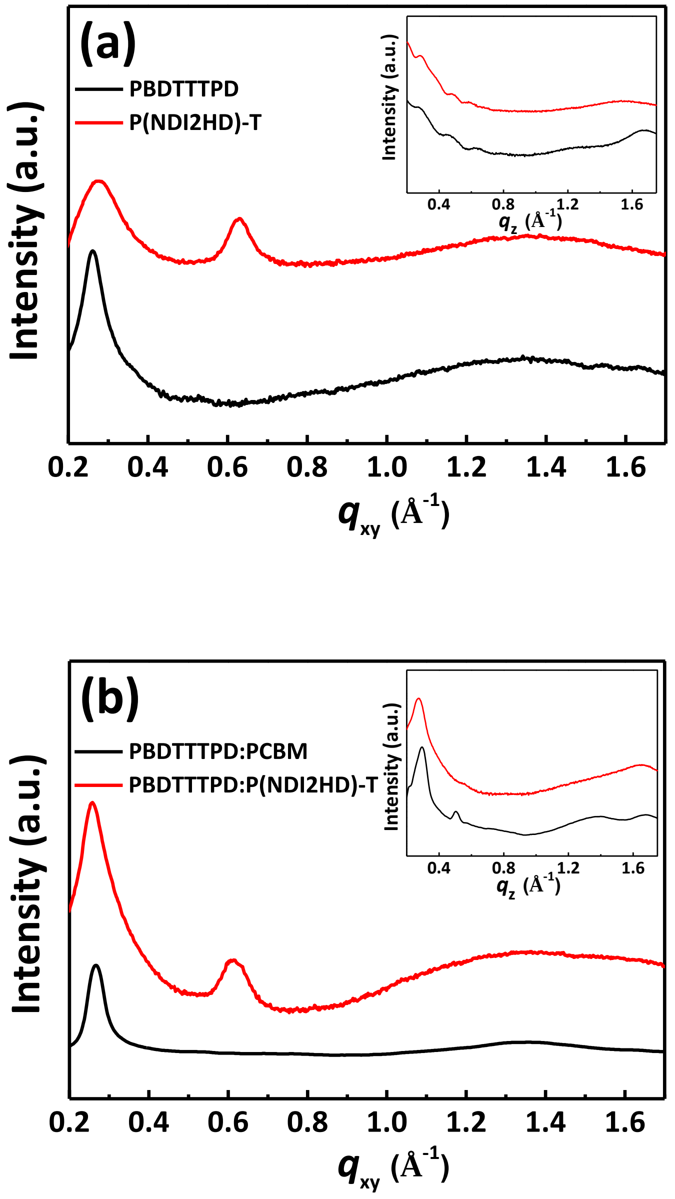

Supplementary Figure 2. Polymer packing structures of pristine polymer and blend films. GIXS in-plane and out-of-plane (inset) line cuts of (a) PBDTTTPD and P(NDI2HD-T) pristine films and (b) PBDTTTPD:PCBM and PBDTTTPD:P(NDI2HD-T) blend films under optimized device condition.

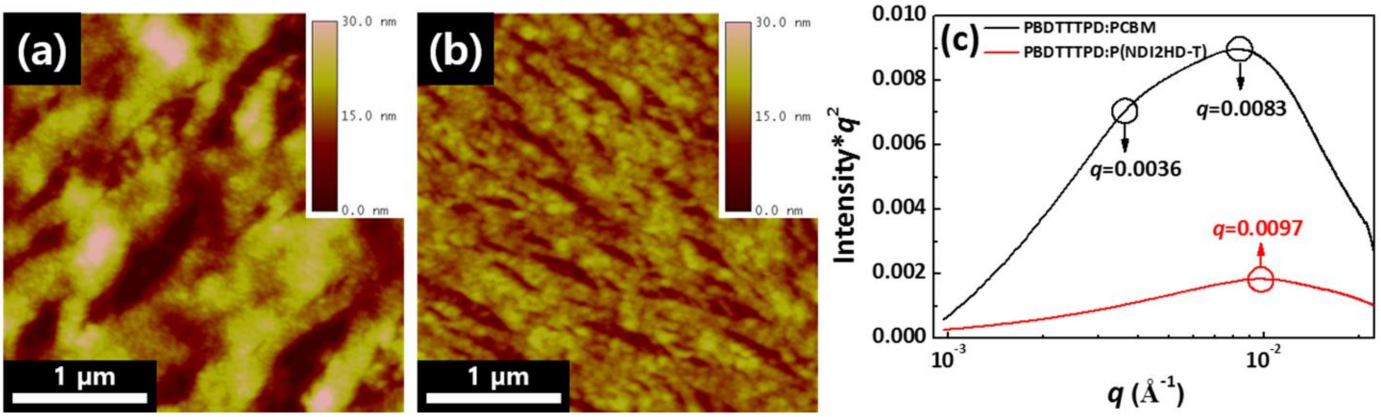

Supplementary Figure 3. Morphological properties of BHJ blend films. AFM height images of (a) PBDTTTPD:PCBM (RMS roughness is 6.4 nm) and (b) PBDTTTPD:P(NDI2HD-T) (RMS roughness is 3.1 nm) blend films under optimized device condition; (c) RSoXS profiles of PBDTTTPD:PCBM and PBDTTTPD:P(NDI2HD-T) blend films (a photon energy at 285.4 eV was used for the maximum scattering contrast between two films).

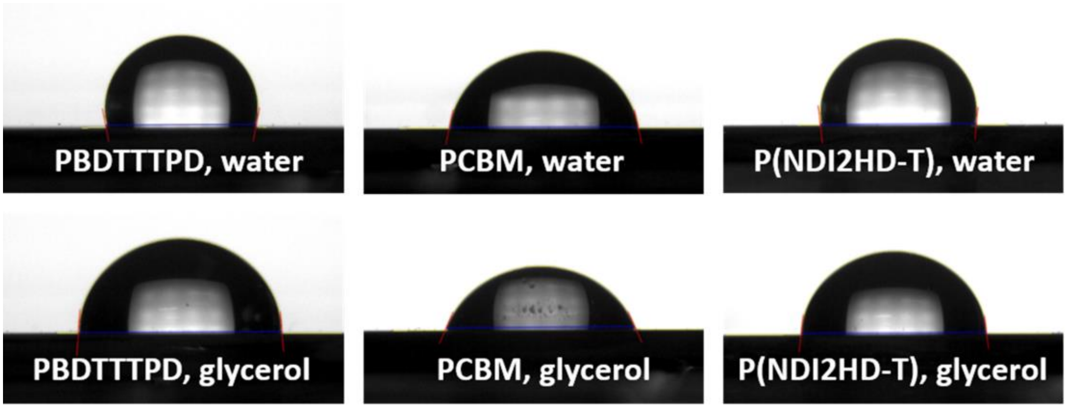

Supplementary Figure 4. Contact angle measurements. Contact angle measurement images of PBDTTTPD, PCBM, and P(NDI2HD-T) neat films by using water and glycerol.

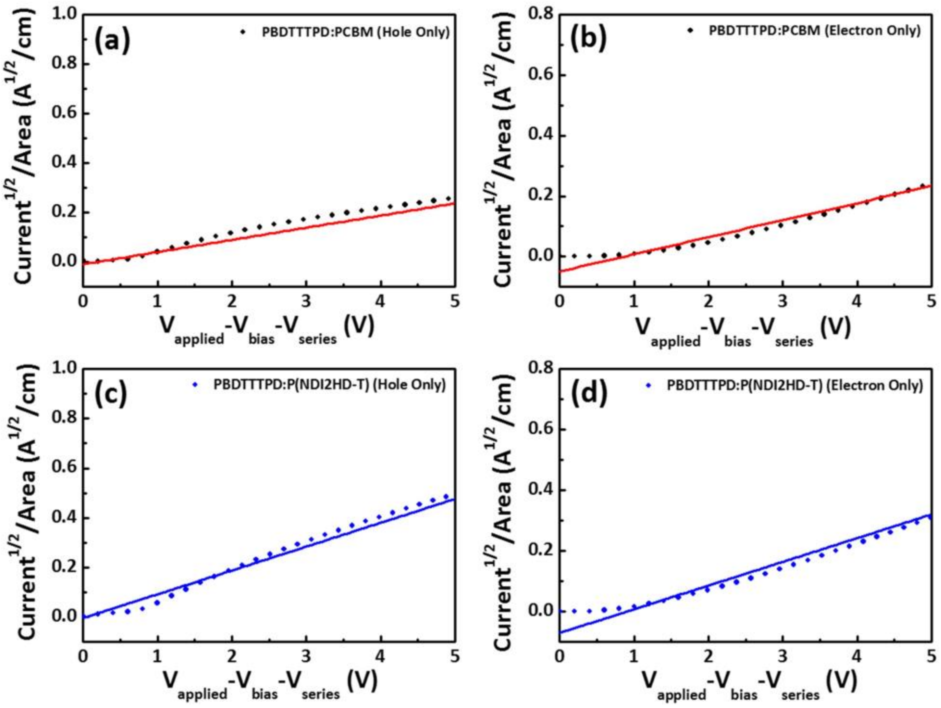

Supplementary Figure 5. Electrical properties of BHJ blend films. Measured space-charge-limited *J*-*V* characteristics of PBDTTTPD:PCBM blends for (a) hole-only and (b) electron-only devices and PBDTTTPD:P(NDI2HD-T) blends for (c) hole-only and (d) electron-only devices under dark conditions.

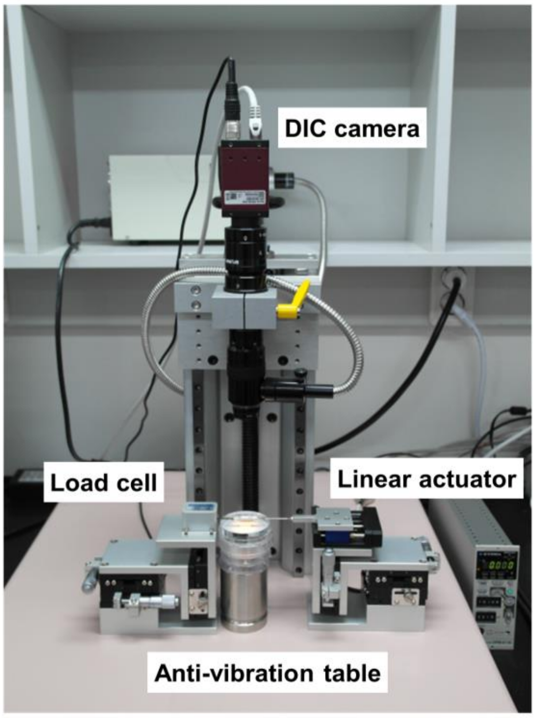

Supplementary Figure 6. Pseudo free-standing tensile testing system. Photograph of the pseudo free-standing tensile testing system. The system consists of a load cell, a linear actuator, and a DIC camera on an anti-vibration table.

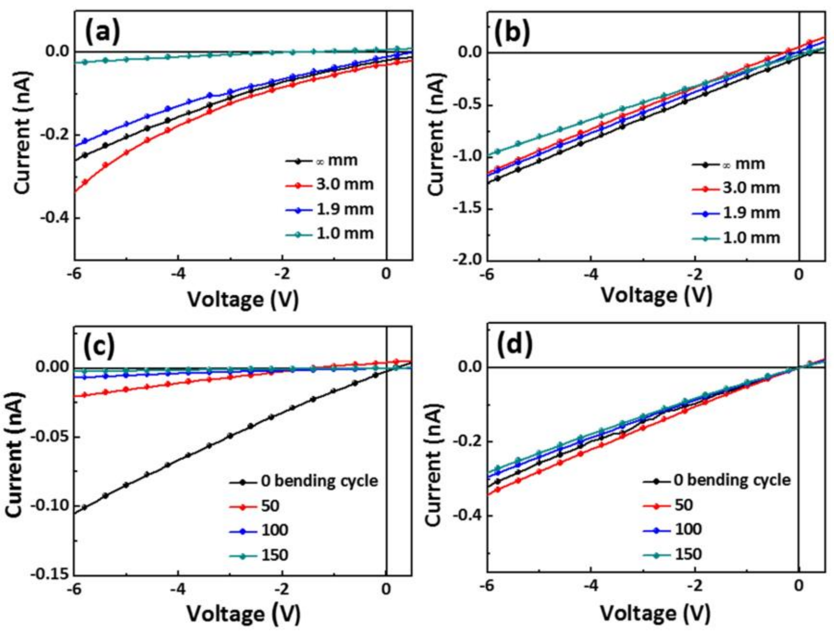

Supplementary Figure 7. Electrical properties of BHJ blend films after bending. *I*-*V* curves of (**a**) PBDTTTPD:PCBM and (**b**) PBDTTTPD:P(NDI2HD-T) blend films measured after bending with respect to different *r* values. *I*-*V* curves of (**c**) PBDTTTPD:PCBM and (**d**) PBDTTTPD:P(NDI2HD-T) blend films measured after multiple cycles of bending at *r* = 1.5 mm.

Supplementary Tables

Supplementary Table 1. Characteristics of PBDTTTPD and P(NDI2HD-T)

**Polymer**	**M_n (kg mol^{-1})^a**	**PDI (M_w/M_n)^a**	**LUMO (eV)^b**	**E_g^{opt} (eV)^c**	**HOMO (eV)**
PBDTTTPD	22	2.02	-3.47	2.02	-5.49
P(NDI2HD-T)	48	2.11	-3.79	1.85	-5.64

[a] The molecular weights and polydispersity index (PDI) of the polymers were determined by GPC using o-DCB as the eluent at 80 °C calibrated by standard PS.[1, 2]
[b] LUMOs were measured by cyclic voltammetry.[1, 2]
[c] Optical bandgaps were determined by UV−vis absorption onsets in the polymer films.[1, 2]

Supplementary Table 2. Detailed photovoltaic parameters of the PBDTTTPD:PCBM based normal type PSC devices with various additive volume fractions

**Additive**	**V_{OC} (V)**	**J_{SC} (mA cm^{-2})**	**FF**	**PCE (%)**
DIO 1 vol%	0.962±0.015	10.021±0.431	0.531±0.009	5.121±0.071
DIO 2 vol%	0.967±0.007	11.050±0.107	0.539±0.001	5.760±0.028
DIO 3 vol%	0.959±0.003	11.208±0.057	0.565±0.007	6.076±0.045
DIO 4 vol%	0.964±0.008	10.289±0.209	0.536±0.007	5.316±0.014

Supplementary Table 3. Detailed photovoltaic parameters of the PBDTTTPD:P(NDI2HD-T) based normal type PSC devices with various additive volume fractions

**Additive**	**V_{OC} (V)**	**J_{SC} (mA cm^{-2})**	**FF**	**PCE (%)**
DIO 0.50 vol%	1.049±0.005	9.831±0.349	0.513±0.010	5.290±0.078
DIO 0.75 vol%	1.060±0.003	10.859±0.097	0.551±0.003	6.348±0.007
DIO 1.00 vol%	1.062±0.001	11.243±0.028	0.553±0.006	6.601±0.058
DIO 1.25 vol%	1.048±0.002	10.436±0.095	0.520±0.006	5.684±0.067

Supplementary Table 4. Detailed photovoltaic parameters of the PBDTTTPD:$PC_{71}BM$ based normal type PSC devices with various additive volume fractions

**Additive**	**V_{OC} (V)**	**J_{SC} (mA cm^{-2})**	**FF**	**PCE (%)**
DIO 1 vol%	0.892	10.755	0.498	4.774
DIO 2 vol%	0.941	11.045	0.528	5.488
DIO 3 vol%	1.001	9.694	0.544	5.281
DIO 4 vol%	0.992	8.819	0.517	4.523

Supplementary Table 5. Contact angles and calculated surface tensions of PBDTTTPD, PCBM, and P(NDI2HD-T); interfacial tensions in PBDTTTPD:PCBM and PBDTTTPD:P(NDI2HD-T)

	**θ_{water} (deg)**	**$\theta_{glycerol}$ (deg)**	**Surface tension (mN m^{-1})**	**Interfacial tension (mN m^{-1})[a]**
PBDTTTPD	100.7	85.5	25.0	-
PCBM	75.2	67.2	34.5	9.0
P(NDI2HD-T)	95.7	82.8	25.2	0.9

[a]The interfacial tension values of PBDTTTPD:PCBM and PBDTTTPD:P(NDI2HD-T).

Supplementary Methods

Materials

2,6-Bis(trimethyltin)-4,8-bis(5-ethylhexyl-2-thienyl)benzo[1,2-b:4,5-b′]dithiophene (BDTT), 1,3-dibromo-5-octylthieno[3,4-*c*]pyrrole-4,6-dione (TPD) and 4,9-dibromo-2,7-bis(2-hexyldecyl)benzo[*lmn*][3,8]phenanthroline-1,3,6,8(2*H*,7*H*)-tetraone were purchased from SunaTech, Inc., and used as received without purification. PCBM was purchased from Nano-C. The 1,8-diiodooctane (DIO) additive was purchased from Aldrich and used as received. As the previous paper reported, the PBDTTTPD donor was synthesized by the microwave-assisted Stille coupling reaction.[1] 2,6-Bis(trimethyltin)-4,8-bis(5-ethylhexyl-2-thienyl)benzo[1,2-*b*:4,5-*b*′]dithiophene (1.0 equiv) and 1,3-dibromo-5-octylthieno[3,4-*c*]pyrrole-4,6-dione (1.0 equiv) were added to a microwave vial equipped with a magnetic bar. Dry chlorobenzene (0.05 M) was injected and the solution was degassed with nitrogen for 20 min. The catalysts of $Pd_2(dba)_3$ (3 mol%) and P(*o*-tolyl)$_3$ (12 mol%) were added, and in sequence, this mixture was degassed for an additional 20 min. The vial was placed in a microwave reactor and stirred at 180 °C for 1 h. After cooling to room temperature, the polymer was precipitated into methanol and filtered through a Soxhlet thimble. The precipitate was purified via Soxhlet extraction sequentially with methanol, hexane, acetone, and dichloromethane. The polymer in the dichloromethane fraction was concentrated under reduced pressure and precipitated into methanol. The polymer was dried under vacuum for 24 h. The P(NDI2HD-T) acceptor was also synthesized via microwave-assisted Stille polycondensation.[2] 2,5-bis(trimethylstannyl)thiophene was synthesized following the previously reported procedures.[2] 4,9-dibromo-2,7-bis(2-hexyldecyl)benzo[*lmn*][3,8]phenanthroline-1,3,6,8(2*H*,7*H*)-tetraone (1.0 equiv) and 2,5-bis(trimethylstannyl)thiophene (1.0 equiv) were added to a microwave vial equipped with a magnetic bar. Dry toluene (0.05 M) was injected and the solution was degassed with nitrogen for 20 min. The catalysts of $Pd_2(dba)_3$ (2 mol%) and P(*o*-tolyl)$_3$ (8 mol%) were added, and in sequence, this mixture was degassed for an additional 20 min. The reaction was performed in a microwave reactor at 150 ˚C for 1 hr. After cooling to room temperature, the resulting gel was diluted with chloroform and precipitated in methanol. The polymer

was purified *via* Soxhlet extraction sequentially with methanol, acetone, hexane, and chloroform. The polymer obtained from the chloroform fraction was precipitated into methanol and finally dried under vacuum for 24 hr.

Preparation of the active layer solutions

Preparation of PBDTTTPD:PCBM solution for normal-type conventional polymer solar cells

PBDTTTPD:PCBM (1:1.5, w/w) blended solution was dissolved in chloroform/1,8-diiodooctane (97 vol% to 3 vol%) and stirred at 45 °C for more than 24 h in a glovebox to ensure complete dissolution of the active materials. The total concentration of the polymer donor in the solution was 10 mg ml^{-1}. The solution was then passed through a 0.45-μm polytetrafluoroethylene syringe filter before use.

Preparation of PBDTTTPD:P(NDI2HD-T) solution for normal-type all-polymer solar cells

PBDTTTPD:P(NDI2HD-T) (1.3:1, w/w) blended solution was dissolved in chloroform/1,8-diiodooctane (99 vol% to 1 vol%) and stirred at 45 °C for more than 24 h in a glovebox to ensure complete dissolution of the active materials. The total concentration of (D+A) in the solution was 12.5 mg ml^{-1}. The solution was then passed through a 0.45-μm polytetrafluoroethylene syringe filter before use.

Space Charge Limited Current (SCLC) measurements

The hole and electron mobilities of the all-polymer blends were measured by the SCLC method using ITO/PEDOT:PSS/polymer blends/Au and ITO/ZnO/polymer blends/LiF/Al devices, respectively. Current-voltage measurements in the range of 0-8 V were taken, and the results were fitted to a space-charge-limited function. The SCLC is described by:

$$J_{SCLC} = \frac{9}{8}\varepsilon\varepsilon_0\mu\frac{V^2}{L^3} \quad (1)$$

where ε_0 is the permittivity of free space (8.85×10^{14} F cm^{-1}), ε is the relative dielectric constant of the active layer (3.2 for PBDTTTPD and P(NDI2HD-T) and 3.9 for fullerene), μ is the mobility of the charge carriers, V is the potential across the device ($V = V_{applied} – V_{bi} – V_r$), and L is the active layer thickness. The series and

contact resistances of the device (15-25 Ω) were measured using blank devices (ITO/PEDOT:PSS/Au and ITO/ZnO/LiF/Al, respectively), and the voltage drop caused by this resistance (V_r) was subtracted from the applied voltage.

Measurements of interfacial tensions of PBDTTTPD:PCBM and PBDTTTPD:P(NDI2HD-T)

Measurements of Surface Tensions of PBDTTTPD, PCBM and P(NDI2HD-T)

The surface tensions of PBDTTTPD, PCBM and P(NDI2HD-T) were calculated using contact angle measurements. The contact angles of two different solvents (water and glycerol) on the PBDTTTPD, PCBM and P(NDI2HD-T) films were measured. The surface tension of each film was calculated with the Wu model and the following equations.[3]

$$\gamma_{\mathrm{water}}(1+\cos\theta_{\mathrm{water}}) = \frac{4\gamma^{\mathrm{d}}_{\mathrm{water}}\gamma^{\mathrm{d}}}{\gamma^{\mathrm{d}}_{\mathrm{water}}+\gamma^{\mathrm{d}}} + \frac{4\gamma^{\mathrm{p}}_{\mathrm{water}}\gamma^{\mathrm{p}}}{\gamma^{\mathrm{p}}_{\mathrm{water}}+\gamma^{\mathrm{p}}} \quad (2)$$

$$\gamma_{\mathrm{glycerol}}(1+\cos\theta_{\mathrm{glycerol}}) = \frac{4\gamma^{\mathrm{d}}_{\mathrm{glycerol}}\gamma^{\mathrm{d}}}{\gamma^{\mathrm{d}}_{\mathrm{glycerol}}+\gamma^{\mathrm{d}}} + \frac{4\gamma^{\mathrm{p}}_{\mathrm{glycerol}}\gamma^{\mathrm{p}}}{\gamma^{\mathrm{p}}_{\mathrm{glycerol}}+\gamma^{\mathrm{p}}} \quad (3)$$

$$\gamma^{\mathrm{total}} = \gamma^{\mathrm{d}} + \gamma^{\mathrm{p}} \quad (4)$$

where γ^{total} is the total surface tension of PBDTTTPD, PCBM and P(NDI2HD-T); γ^{d} and γ^{p} are the dispersion and polar components of γ^{total}, respectively; γ_i is the total surface tension of material i, where i = water or glycerol; γ_i^{d} and γ_i^{p} are the dispersion and polar components of γ_i, respectively, and θ is the contact angle of the droplet (water or glycerol) on the PBDTTTPD, PCBM and P(NDI2HD-T) films.

In addition, to elucidate the relationship between the miscibility and the performances of the PSCs, the interfacial tension between PBDTTTPD and PCBM, and PBDTTTPD and P(NDI2HD-T) were calculated using the following equation.[4]

$$\gamma_{12} = \gamma_1 + \gamma_2 - \frac{4\gamma_1^{\mathrm{d}}\gamma_2^{\mathrm{d}}}{\gamma_1^{\mathrm{d}}+\gamma_2^{\mathrm{d}}} - \frac{4\gamma_1^{\mathrm{p}}\gamma_2^{\mathrm{p}}}{\gamma_1^{\mathrm{p}}+\gamma_2^{\mathrm{p}}} \quad (5)$$

where γ_{12} is the interfacial tension between PBDTTTPD (1) and PCBM (2) (or PBDTTTPD (1) and P(NDI2HD-T) (2)); γ_j is the surface tension of material j, where j = 1 or 2, and the dispersion and polar

components of γ_j are denoted as γ_j^{d} and γ_j^{p} that are calculated using the contact angle of the water and glycerol droplet on the PBDTTTPD, PCBM and P(NDI2HD-T) films.

Supplementary References

1. Kang, T.E. *et al.* Importance of Optimal Composition in Random Terpolymer-Based Polymer Solar Cells. *Macromolecules* **46**, 6806-6813 (2013).
2. Lee, C. *et al.* High-Performance All-Polymer Solar Cells Via Side-Chain Engineering of the Polymer Acceptor: The Importance of the Polymer Packing Structure and the Nanoscale Blend Morphology. *Adv. Mater.* DOI: 10.1002/adma.201405226 (2015).
3. Comyn, J. Contact angles and adhesive bonding. *Int. J. Adhes. Adhes.* **12**, 145-149 (1992).
4. Wu, S. Calculation of interfacial tension in polymer systems. *J. Polym. Sci., Polym. Symp.* **34**, 19-30 (1971).
